# Magnetic resonance imaging of Huntington's disease: preparing for clinical trials

**DOI:** 10.1016/j.neuroscience.2009.01.045

**Published:** 2009-11-24

**Authors:** S. Klöppel, S.M. Henley, N.Z. Hobbs, R.C. Wolf, J. Kassubek, S.J. Tabrizi, R.S.J. Frackowiak

**Affiliations:** a Department of Psychiatry and Psychotherapy, Freiburg Brain Imaging, University Clinic Freiburg, Hauptstrasse, 79108 Freiburg, Germany; b Dementia Research Centre, Institute of Neurology, University College London, Queen Square, London WC1N 3BG, UK; c Department of Psychiatry and Psychotherapy III, University of Ulm, Leimgrubenweg, 89075 Ulm, Germany; d Department of Neurology, University of Ulm, Oberer Eselsberg, 89081 Ulm, Germany; e Department of Neurodegenerative Disease, Institute of Neurology, University College London, Queen Square, London WC1N 3BG, UK; f Wellcome Trust Centre for Neuroimaging, Institute of Neurology, University College London, Queen Square, London WC1N 3BG, UK; g Département d'études cognitives, Ecole Normale Supérieure, 29 rue d'Ulm, Paris 75005, France; h Laboratory of Neuroimaging, IRCCS Santa Lucia, Via Ardeatina, Roma 00179, Italy

**Keywords:** Huntington's disease, imaging, basal ganglia, subject stratification, AD, Alzheimer's disease, BOLD, blood-oxygenation level dependent, BSI, brain boundary shift integral, CSF, cerebrospinal fluid, DWI, diffusion-weighted imaging, FA, fractional anisotropy, fMRI, functional magnetic resonance imaging, FWHM, full-width at half-maximum, GM, grey matter, HD, Huntington's disease, ICA, independent component analysis, MRI, magnetic resonance imaging, PD, Parkinson's disease, PSC, presymptomatic gene carriers, ROI, region of interest, TCN, temporally coherent networks, TIV, total intracranial volume, VBM, voxel-based morphometry, WM, white matter

## Abstract

The known genetic mutation causing Huntington's disease (HD) makes this disease an important model to study links between gene and brain function. An autosomal dominant family history and the availability of a sensitive and specific genetic test allow pre-clinical diagnosis many years before the onset of any typical clinical signs. This review summarizes recent magnetic resonance imaging (MRI)–based findings in HD with a focus on the requirements if imaging is to be used in treatment trials. Despite its monogenetic cause, HD presents with a range of clinical manifestations, not explained by variation in the number of CAG repeats in the affected population. Neuroimaging studies have revealed a complex pattern of structural and functional changes affecting widespread cortical and subcortical regions far beyond the confines of the striatal degeneration that characterizes this disorder. Besides striatal dysfunction, functional imaging studies have reported a variable pattern of increased and decreased activation in cortical regions in both pre-clinical and clinically manifest HD-gene mutation carriers. Beyond regional brain activation changes, evidence from functional and diffusion-weighted MRI further suggests disrupted connectivity between corticocortical and corticostriatal areas. However, substantial inconsistencies with respect to structural and functional changes have been reported in a number of studies. Possible explanations include methodological factors and differences in study samples. There may also be biological explanations but these are poorly characterized and understood at present. Additional insights into this phenotypic variability derived from study of mouse models are presented to explore this phenomenon.

Huntington's disease (HD) is an autosomal dominant neurodegenerative disorder with an average age of onset around 40 years and an incidence of ∼4–10 per 100,000 in the United States and Western European countries. It is caused by a CAG repeat expansion in the gene encoding the protein huntingtin. The availability of a sensitive and specific genetic test allows pre-clinical diagnosis, many years before the onset of unequivocal motor signs (Walker, 2007).

Within the present overview, we plan to update and extend recent review articles on imaging in HD (Henley et al., 2005; Montoya et al., 2006; Aylward, 2007; Bohanna et al., 2008). We will focus on the use of imaging for clinical therapeutic trials and will consider factors that need to be taken into account when designing them. In a disease like HD, there is a need to follow the effects of neuro-degeneration in individuals over time to evaluate degeneration-modifying treatments. Optimally, monitoring should occur in both the pre-clinical and clinically manifest stages of the disease. Currently, it is difficult to monitor the progression of neuro-degeneration in asymptomatic individuals using clinical or neuropsychological testing. Ideally, imaging would be used to stratify subjects by degree of caudate atrophy, well before the onset of unequivocal clinical signs. Such stratification would potentially make it possible to stage subjects into more homogeneous populations and to assess the effects of treatments on different stages in the evolution of this neuro-degenerative disorder.

We will focus mainly on T1-weighted structural imaging methods since treatment trials are likely to include multiple clinical centers where technical requirements for diffusion weighted imaging (DWI) or functional magnetic resonance imaging (fMRI) studies are often not readily available and potentially more difficult to standardize. There are however first encouraging data from DWI (Landman et al., 2007; Wakana et al., 2007) and fMRI studies (Bosnell et al., 2008) preparing for multi-center and multimodality strategies and applications in the future.

We start by briefly outlining structural imaging methods, including some caveats in their application. In the second section, we highlight current evidence for structural imaging abnormalities in HD, associations between atrophy visualized *in vivo* and clinical performance, and discuss imaging evidence for the stratification of HD subjects based on, e.g. the number of CAG repeats, clinical phenotype, or pattern of cognitive impairment. We consider this critical for the optimal design of clinical trials, as clinically defined characteristics may be associated with differential rates and distribution of degeneration. The third and fourth section reviews fMRI and DWI data with a focus on their usefulness for the characterization of individual subjects before entering treatment trials. The fifth section summarizes data from imaging studies in HD mouse models and illustrates how such data aid in the stratification of subjects and in the interpretation of imaging findings.

Beyond issues related to imaging-based criteria for subject inclusion in trials, the next step will be to find sensitive methods to detect possible effects of proposed treatments. The concluding section therefore provides an update on the ongoing discussion about imaging-derived measures as biomarkers of disease progression.

This selective review will restrict itself to magnetic resonance imaging (MRI). Imaging of cerebral blood flow and glucose metabolism has been the focus of another recent review (Ma and Eidelberg, 2007).

## Structural imaging methods

Structural MRI analyses in HD have yielded inconsistent findings (see 2nd section). Discrepancies are likely to be due to methodological differences in the analysis techniques applied, as well as true heterogeneity in the cohorts studied. In this section we summarize some of the image analysis techniques currently used to investigate neurodegeneration in HD.

Structural MRI investigations typically fall into two categories: (1) hypothesis-driven region of interest (ROI) studies on pathologically-affected structures, and (2) more exploratory unbiased whole-brain analyses, requiring no a priori assumptions.

### ROI analyses

The current gold-standard measurement technique for ROI volumetry is manual segmentation. This involves an expert rater tracing around the outline of the structure on every “slice” of the MR image. Manual segmentation has been used extensively to investigate atrophy in the caudate, putamen, frontal lobe, and globus pallidus (Harris et al., 1992; Aylward et al., 1997, 1998; Aylward et al., 2000; Rosas et al., 2001, 2003; Fennema-Notestine et al., 2004; Paulsen et al., 2006b). However, this approach is extremely time consuming, and results are subject to inter- and intra-rater variability.

As an alternative to manual-outlining algorithms, several fully automated segmentation and anatomical labeling techniques have been developed (Khan et al., 2008). These techniques are likely to be used more frequently in future studies if they are shown to be reliable. Both manual- and automated-ROI-based analyses are limited by difficulties in accurately and reliably outlining small structures, especially if the structure is complex and contains poorly defined boundaries.

### Whole-brain analyses

The most elementary whole-brain analysis is visual assessment of the MR images by a radiologist. However, quantification via visual rating scales is crude and subject to inter- and intra-rater variation.

The brain boundary shift (BSI) integral is a semi-automated technique for measuring whole-brain volume change from serial MRI (Freeborough and Fox, 1997), and can be used to capture the full extent of diffuse cerebral volume loss in HD (Henley et al., 2006). The volume of atrophy is measured directly from the difference in brain boundaries of registered scan pairs, thereby, reducing the impact of segmenter variability on results since the quantification does not rely on manual segmentation per se. One limitation of the BSI is its inability to localize change to specific tissues. In addition, there is a possibility that degeneration in one structure might be accompanied by an enlargement in another which may not result in changes at the brain boundary.

Voxel-based morphometry (VBM) (Ashburner and Friston, 2000) and cortical thickness analyses (Fischl and Dale, 2000; Lerch et al., 2008a) are unbiased whole-brain-based methods used to study the pattern of neurodegeneration and neuro-anatomical correlates of HD. Although these methods are automated, there are numerous differences between research groups in the software used, the processing steps and statistical models applied, and the correction of the resulting statistical parametric maps, all of which may affect overall results. A principled approach is therefore mandatory.

In brief, VBM requires a series of automated image pre-processing steps, including segmenting images into grey matter (GM), white matter (WM) and cerebrospinal fluid (CSF) compartments, spatially aligning (normalizing) these compartments into standard stereotactic space and applying appropriate smoothing for subsequent analysis at group level. A number of different analytical path specifications have been used and reported: some studies optimize the normalization but not segmentation, some use a study-specific template for normalization and these may be whole brain templates or templates designed for the tissue classes under study. Some researchers introduce other new steps into the analytic path with varying degrees of a priori justification (compare, e.g. Thieben et al., 2002; Peinemann et al., 2005; Douaud et al., 2006).

Within the few published cortical thickness analyses in HD, there is variation in both the software and methodology applied. Rosas et al. (e.g. Rosas et al., 2008) use the surface-based software package FreeSurfer (Fischl and Dale, 2000), which automatically extracts the inner and outer cortical surfaces using deformable models, and calculates cortical thickness as the shortest distance between the pial and WM surfaces (Fischl and Dale, 2000). By contrast, cortical thickness measurements in the YAC128 mouse-model study (see further below) use Laplace's equation to create streamlines between the inside and outside cortical surfaces, with the length of streamline giving the measurement of thickness (Lerch et al., 2008a). Nopoulos et al. (2007) used the BRAINS2 family of software programs (Magnotta et al., 2002) to extract a model representing the parametric center of the GM tissue class, which approximates the spatial center of the cortex. Here, cortical depth is measured as the minimum distance between the 100% GM surface and the center of the cortex (i.e. 50%/50% GM/WM surface). These methodological differences are likely to have some effect on results and should be considered when comparing studies of cortical thickness.

There is a large amount of inconsistency in the specification of the covariates included in statistical models for analysis (O'Brien et al., 2006). VBM studies often, but not always, include age, gender, and total intracranial volume (TIV) as nuisance factors, since they can affect brain volume independently of disease. VBM studies also tend to adjust for total GM volume. This approach allows for investigation of region-specific patterns of atrophy and relative atrophy or preservation of tissue (Muhlau et al., 2007); however it is important that GM volume is not used as a substitute for head size, particularly in studies of neurodegeneration, as total as well as regional GM volume may vary with disease severity and duration. Cortical thickness. Cortical thickness analyses tend to correct for age and gender but not TIV. There appears to be no relationship between brain size and cortical thickness in mice (Lerch et al., 2008a) but recent work indicates that the relative dimensions of sulcal depth with respect to cortical thickness change with head size in humans (Im et al., 2008).

**Fig. 1:**
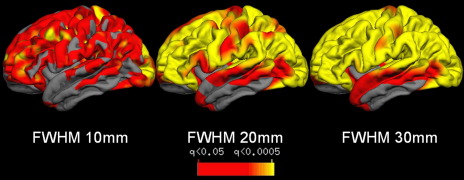
The figure illustrates the impact of different smoothing kernels (ranging from 10 to 30 mm) on the apparent distribution and severity of cortical thinning.

Some smoothing is generally required to compensate for small variation in individual anatomy during inter-subject registration, and to ensure that the data are normally distributed (statistically stationary). The amount of smoothing sensitizes analyses to a particular spatial scale of effect (Ridgway et al., 2008) which will affect interpretation of results; the detection of change in a structure is more sensitive if the smoothing kernel approximates the size of the structure. For VBM analyses, segments are smoothed through convolution with a volumetric gaussian kernel. Kernel sizes in the HD literature range from full-width at half-maximum (FWHM) 4 mm (Barrios et al., 2007) to 12 mm (Kipps et al., 2005). However, the majority of studies do not justify their choice of smoothing kernel, for example, by attempting to match the spatial extent to that of the degenerative change expected (see Kassubek et al., 2004b for an example of selecting the smoothing kernel based on visual inspection of structural changes). In cortical thickness analyses, smoothing is typically carried out across the cortical surface to follow the topology of the cortex (Rosas et al., 2002, 2005, 2008; Lerch et al., 2008a). The amount of smoothing varies greatly in the few cortical papers published, for example Rosas et al. (2005) used a 2D gaussian kernel of FWHM 29 mm but a 3D kernel of FWHM 16 mm in earlier work. Fig. 1 shows the effects of varying a surface-based gaussian smoothing kernel from FWHM 10–30 mm on the same dataset. As the kernel size increases, the area of significant differences tends to increase. Therefore, differences between studies using different smoothing kernel types may not reflect true disease-related differences in the cohorts studied. For example, Lerch et al. (2008a) found an increase in cortical thickness of the sensorimotor cortex in the YAC128 mouse HD model relative to wild-type mice at a stage representing very early stage HD/presymptomatic gene carriers (PSC). This finding agrees with some results from human PSC (Paulsen et al., 2006b), but contradicts others; Rosas et al. (2005) found regionally-selective thinning and no areas of thickening in their PSC cohort.

In summary, this section has illustrated a number of methodological issues which are likely to influence imaging findings. However, it is difficult to predict how each issue will affect the overall result. The effects of smoothing-kernel size have been explored systematically (Jones et al., 2005) but are likely to be dependent on the specific study. In contrast, the effect of changing regressors in the statistical design or using different means for spatial normalization is very difficult to predict. Such methodological issues need to be considered when trying to interpret the HD literature and to generalize results. Ideally, as has been suggested recently (Nopoulos et al., 2007), researchers need to consolidate methods in order to produce findings that are replicable, robust, and truly representative of HD. Recently published sets of guidelines (Ridgway et al., 2008) could prove helpful in that respect. Such general guidelines are preferable to very specific ones (e.g. a specific smoothing kernel or a specific set of covariates) as their choice will depend on the exact research question and dataset being investigated.

## Structural correlates of clinical features and subject stratification

In this section, we summarize recent structural MRI work in HD, as well as how these *in vivo* findings relate to the clinical features observed in disease. We also highlight evidence that stratification based on e.g. number of CAG repeats or clinical phenotype, yields distinct patterns of atrophy. Tables providing an excellent overview of previously published studies are provided in another review (Bohanna et al., 2008).

### Structural MRI in HD

**Fig. 2:**
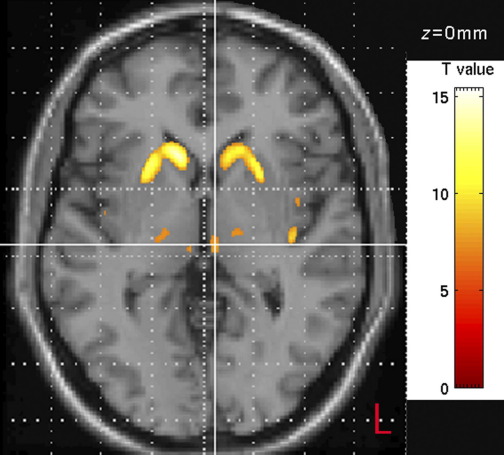
Example displays areas with decreased GM (*P*<0.001) as detected by VBM comparing early HD subjects to controls (see Kassubek et al., 2005; for details on group characteristics and methodology).

The most striking pathological changes in HD occur in the striatum (Halliday et al., 1998; Gutekunst et al., 2002) and there is a wealth of evidence that striatal atrophy can be detected using MRI up to 23 years prior to predicted motor onset and that striatal volume is negatively correlated with motor and cognitive function and CAG repeat length (Harris et al., 1992, 1996, 1999; Aylward et al., 1994, 1996, 2004; Rosas et al., 2001; Kassubek et al., 2004b; Paulsen et al., 2006b; Fig. 2).

The contribution of cortical regions to the pathophysiology of HD has been of increasing interest, since huntingtin-protein aggregates have been found to concentrate in cortical neurons to an even greater extent than in the striatum (Ferrante et al., 1997; Sapp et al., 1997; Gutekunst et al., 1999). Extra-striatal atrophy has been reported in early HD including WM loss in the absence of total brain volume or GM loss (Aylward et al., 1998; Beglinger et al., 2005). Others have reported reduced total brain volume or GM loss (Rosas et al., 2003; Fennema-Notestine et al., 2004; Kassubek et al., 2004a,b).

Reduced cortical thickness has been found in early to moderate HD, with a suggestion that the sensorimotor areas are most severely affected and that atrophy progresses from posterior to anterior regions with longer disease duration (Rosas et al., 2002, 2008). Of studies taking a whole-brain rather than an ROI approach, most find evidence of bilateral caudate and putaminal atrophy in both early disease and in PSC (Thieben et al., 2002; Peinemann et al., 2005). In contrast there is less consensus about extra-striatal atrophy; for example insular atrophy has been shown in PSC (Thieben et al., 2002), but not in a study in early HD (Kassubek et al., 2004b). There are similarly variable results on the involvement of the thalamus, amygdala, hypothalamus, and frontal areas (Kassubek et al., 2004b, 2005; Peinemann et al., 2005; Douaud et al., 2006; Muhlau et al., 2007). This could be an effect of relatively small group sizes, different clinical stages or the choice of statistical thresholds. It should also be kept in mind that increasing levels of degeneration can result in problems with image registration in areas not themselves affected by disease (e.g. an inward shift of the insular cortex with shrinking striatal volume).

Abnormal brain volume has also been found in PSC. A recent study found evidence of reduced WM but normal GM volume in PSC approximately 11 years from predicted clinical onset (Paulsen et al., 2006b). In contrast, PSC estimated at 23 years from onset had more grey and less WM than normal, in the absence of any increase in ventricular size or CSF volume, which led the authors to postulate that their result may represent a developmental abnormality rather than a very early neurodegenerative process (Paulsen et al., 2006b). Total brain volume was not significantly reduced although further work on the same cohort demonstrated morphological GM abnormalities, including increased gyral surface area, thicker gyri, and thinner sulci (Nopoulos et al., 2007). Decreased WM has been reported to correlate with years to onset in PSC, while the same study showed a non-significant reduction of GM (Ciarmiello et al., 2006). However, GM thinning in regions across the cortex has also been reported in this population (Rosas et al., 2005).

In summary, structural MRI studies in early HD tend to support the postmortem findings (Vonsattel and DiFiglia, 1998; Gutekunst et al., 2002) of widespread generalized atrophy throughout the cortex, with disproportionate volume loss in the striatum. Where both GM and WM are measured, there is a suggestion that WM loss is relatively greater or seen earlier in the course of the degeneration (see section on DWI). PSC have clear striatal atrophy and there is evidence of WM loss but the involvement of cortical GM is less clear. There is considerable variation between studies both with regard to the extent of extra-striatal atrophy and the point in the disease process at which loss in different regions starts to become significant. In many cases these differences may be due to methodological differences or lack of statistical power caused by the small or heterogeneous groups selected for study. Alternatively, they may reflect true heterogeneity in the populations sampled. Although it is clear that widespread structural loss occurs early in the disease, more work is needed to define the pattern, time course, severity, significance, and evolution of these changes more clearly, particularly in the early PSC phase.

The majority of longitudinal structural MRI studies in HD have focused on the striatum and it is now well established that striatal atrophy rates are increased prior to onset of motor symptoms and that they may not be linearly progressive, at least in the pre-clinical stage. Atrophy rate is also correlated with CAG repeat length (Aylward et al., 1997, 2000, 2004; Aylward, 2007). Other studies focusing on the whole brain have reported a decrease in WM volume in PSC over periods as short as 16 months (Ciarmiello et al., 2006), but others have reported atrophy of subcortical GM but no WM changes in PSC subjects over a period of 2 years (Kipps et al., 2005). In clinically manifest HD, there have been reports of no change over 18 months (Ciarmiello et al., 2006), increased whole-brain atrophy rates measured over 6 months (Henley et al., 2006), and a decrease in insula, cerebellum and some regional cortical volumes over a 1-year period (Ruocco et al., 2008). The specificity of the latter results may need clarification as subjects were aged from 4 to 73 years, but results were not adjusted for either age or head-size (Ruocco et al., 2008).

### Neural correlates of clinical performance in HD

A number of imaging studies have confirmed the existence of a relationship between striatal volumes and cognitive deficits (Bamford et al., 1989; Starkstein et al., 1992; Brandt et al., 1995; Harris et al., 1996). Measures of frontal lobe size correlate with memory and planning performance (Bamford et al., 1989, 1995; Backman et al., 1997) and regions including the thalamus (Kassubek et al., 2005), insula (Peinemann et al., 2005), WM (Beglinger et al., 2005; Rosas et al., 2006), and widespread cortical regions (Rosas et al., 2005, 2008) are associated with cognitive performance in both PSC and early clinically manifest HD. Most of these studies have focused on a few tests in a specific domain (executive function) although recently it has also been shown that striatal and frontal regions are associated with performance on emotion recognition (Hennenlotter et al., 2004; Kipps et al., 2007; Henley et al., 2008).

### Stratification of subjects for clinical trials

A problem in HD research is the heterogeneity of subject groups, making it hard to detect small differences in variables of interest or to track change over time. One solution is to stratify subjects based on clinical characteristics, and it has been demonstrated that such stratified groups do show different patterns of atrophy. When stratified by CAG repeat length, motor or cognitive score, subjects with longer repeat lengths (or worse scores) show more extensive atrophy, that also affects extra-striatal regions, than those with shorter repeat lengths (or better motor scores) (Kassubek et al., 2004b), when compared with controls. PSC subjects, when stratified by estimated time to clinical onset, also demonstrate differences in the amount of striatal, grey and WM atrophy that are time dependent (Paulsen et al., 2006a). More recently, there has been a suggestion that patients showing more bradykinetic features of HD show a different pattern of cortical thinning to those without these features (Rosas et al., 2008). However, in most analyses of this type small subgroups were compared with controls, rather than directly with each other. Therefore, more work is needed to validate MRI differences and to determine whether they can be used to stratify pre-clinical and symptomatic subjects so that more homogeneous cohorts can be entered into clinical trials.

## Functional MRI in HD

Within the past 5 years, fMRI has been increasingly used to study basic sensory, emotional and cognitive processes in HD. In this section we seek to update the reader on new developments in fMRI research since the review recently provided by Bohanna et al.(2008) and address three main questions that arise from published fMRI findings in clinical HD and PSC.

### Does evidence from fMRI suggest a distinct pattern of neural dysfunction during cognitive processing in HD?

Published fMRI studies (see Bohanna et al., 2008 for tables providing an overview of the main findings) have shown that abnormal activation patterns including hyper- and hypo-activation in HD patients and PSC are not restricted to the striatum. The stage of the disease as well as the clinical phenotype might contribute to data heterogeneity, and differential patterns of neural dysfunction during different stages of the illness still have to be determined in larger patient cohorts. Taking these clinical limitations into account, the extant fMRI data in clinical HD suggest a complex pattern of neural dysfunction during cognitive processing, including a widespread set of subcortical and cortical regions which have been found to show abnormal brain activation responses compared to healthy subjects. These findings, however, do not converge to define a single functional biomarker of HD, but rather suggest a dynamic pattern of neural dysfunction in clinical HD and PSC which possibly depend on task type, cognitive demand, task performance, or a combination of all three variables.

For instance, abnormal activation of the anterior cingulate cortex in clinical HD has been shown during the Simon response conflict task (Georgiou-Karistianis et al., 2007), but not during processing by working memory (Wolf et al., 2008c) or during implicit motor learning (Kim et al., 2004). These results suggest that the detection threshold for aberrant neural responses in the cingulate cortex could be task-dependent. Furthermore, although the majority of fMRI studies have so far demonstrated reduced task performance during cognitive processing in clinical HD (Kim et al., 2004; Georgiou-Karistianis et al., 2007; Wolf et al., 2008c), the relationship between task performance and brain dysfunction has to be determined, since both decreased brain activation (Wolf et al., 2008c) and increased functional connectivity (Thiruvady et al., 2007) in clinical HD have been discussed against the background of impaired task accuracy. Several authors have discussed cortical hyperactivation shown by fMRI in terms of neural “compensation” (Paulsen et al., 2004; Georgiou-Karistianis et al., 2007; Wolf et al., 2007; Zimbelman et al., 2007), although the compensatory processes related to functional abnormalities remains to be characterized in terms of a task-related mechanism. Indeed, increased brain activation in the presence of task-stimulation does not necessarily have to mirror task-specific processes, and thus other explanations appear plausible, e.g. the possibility of abnormally increased activation that interferes with brain activation typically elicited by a given task. For instance, a positive correlation between the UHDRS motor score and activation of right dorsal and left ventral premotor regions has been reported in clinically affected patients with HD while performing a response conflict task (Georgiou-Karistianis et al., 2007). The authors interpreted this relationship as related to characteristics of the disease, and suggested that patients with more severe motor symptoms could exhibit greater baseline motor region activity in order to inhibit involuntary movements, while the alternative interpretation of a relationship between premotor activation and increasing task difficulty needs further validation.

Moreover, the question is open whether brain activation abnormalities during cognitive performance as shown in HD are qualitatively different from the pattern of neural dysfunction observed in other basal ganglia disorders, e.g. Parkinson's disease (PD) (Monchi et al., 2000; Lewis et al., 2003). For instance, reduced activation of the prefrontal cortex and the putamen during working memory performance has been reported in both patients with PD (Lewis et al., 2003) and HD (Wolf et al., 2008c). Furthermore, somewhat similar to fMRI findings in HD, patterns of both increased and decreased brain activation have been previously reported in PD patients during executive processing (Monchi et al., 2007). While the notion of a differential engagement of the caudate nucleus during distinct processing phases of a cognitive task (Monchi et al., 2006, 2007) could partially explain divergent findings of increased and reduced brain activation in HD and PSC, this hypothesis clearly needs to be explicitly tested in HD patients and PSC.

Eventually, it is also unclear if increased brain activation in HD patients and PSC could also reflect increased cortical “noise” and thus a loss of regional specialization (Rajah and D'Esposito, 2005), e.g. due to a generalized dopaminergic deficit, rather than reflecting circumscribed deficits due to the disease or due to task-specific factors. Furthermore, the impact of GM volume changes and abnormal neurovascular coupling on neural activation and blood-oxygenation level dependent (BOLD) responses is not well-known yet, and could additionally lead to aberrant BOLD fluctuations associated with neurodegeneration and cerebrovascular dysregulation (Deckel et al., 1998; Deckel, 2001).

### Can functional connectivity analyses provide further insights into early pathophysiological processes in PSC and into disease-related brain dysfunction during the symptomatic period?

Given the association between striatal abnormalities and cognitive performance in PSC (Backman et al., 1997; Solomon et al., 2007), as well as anatomical evidence for topographically organized reciprocal connections between the striatum and neocortex (Middleton and Strick, 2000), it has been suggested that dysfunction of corticostriatal circuits may characterize pre-clinical stages of HD (Reading et al., 2004; Feigin et al., 2006) before the onset of overt brain atrophy and motor, psychiatric or cognitive disturbances.

**Fig. 3:**
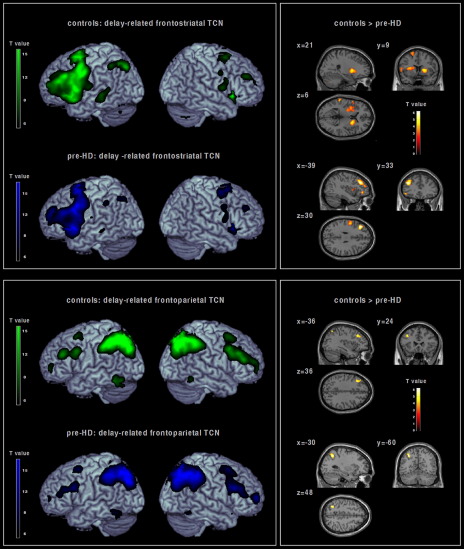
Positive TCN identified by means of ICA in healthy controls (green) and pre-HD subjects (blue) during the delay period of a verbal working memory task (Wolf and Walter, 2005; Wolf et al., 2008a). Top right: Within a predominantly frontostriatal TCN, areas with decreased connectivity in pre-HD individuals versus healthy comparison subjects comprised the left ventro- and dorsolateral prefrontal cortex, the left parietal lobule, the left insula, the bilateral putamen and the right caudate. Bottom right: Within a predominantly frontoparietal TCN, areas with decreased connectivity in pre-HD individuals versus healthy comparison subjects comprised the left dorsolateral prefrontal cortex and the left superior parietal cortex (Wolf et al., 2008b). The 2nd level spatial maps are rendered on the anatomical templates implemented in MRIcron [http://www.sph.sc.edu/comd/rorden/mricron/] (left) and SPM5 (right).

It is unclear at present if regionally abnormal brain activation during the presymptomatic period reflects aberrant corticostriatal pathways related to progressive subcortical atrophy, or if a primarily cortical deficit independent of the neurodegenerative process in the striatum could additionally account for findings of primarily cortical dysfunction in PSC (Reading et al., 2005; Wolf et al., 2007). Although it is neuroanatomically plausible that cortical regions showing abnormal activation in PSC could reflect an early disruption of corticostriatal networks, e.g. as a consequence of impaired corticostriatal connectivity, recent evidence suggests that aberrant connectivity of functionally related networks extends beyond corticostriatal decoupling. For instance, a lower expression of functional networks involving dissociable dorsolateral prefrontal circuits was found in PSC during working memory processing despite absent differences in task performance compared to healthy controls (Wolf et al., 2008a). By means of a model-free multivariate analysis technique (independent component analyses; ICA), two distinct temporally coherent networks (TCNs) (Calhoun et al., 2008a) were identified that were positively correlated with the delay period of a verbal working memory paradigm. These TCNs comprised cortical and subcortical regions specified by a distinct spatiotemporal pattern comprising frontostriatal and fronto-parietal areas (Fig. 3). In PSC, decreased functional connectivity was found in left lateral prefrontal and bilateral striatal areas, as well as in left fronto-parietal regions compared to healthy controls. Interestingly, contributions of the lateral prefrontal cortex were not confined to frontostriatal pathways, but were also detected in left lateralized fronto-parietal networks with sparing of the striatum, suggesting additional cortical decoupling without involvement of subcortical structures (Wolf et al., 2008a). It is yet an open question however, whether impaired striatal transmission can also explain the latter findings since multimodal imaging data (e.g. PET and fMRI measures obtained in an identical PSC sample) are lacking at present.

Regionally increased activation of the anterior cingulate cortex (Georgiou-Karistianis et al., 2007), as well as disrupted functional connectivity between the anterior cingulate and lateral prefrontal cortex has been shown in clinically affected subjects. The latter finding was associated with slower reaction times and more frequent errors during a Simon task (Thiruvady et al., 2007). These findings are suggestive of a functional decoupling between the anterior cingulate and the lateral prefrontal cortex in HD patients, which might partly account for the deterioration in performance. Increased cortical activation could mirror regional compensatory processes as a result of this impaired connectivity (Thiruvady et al., 2007). In contrast to these findings, impaired brain activation in the absence of coincidental hyper-activation has been found during working memory processing at intermediate and high levels of cognitive processing (Wolf et al., 2007). One possible explanation for these findings is related to load-dependent mechanisms of frontostriatal function, which have been repeatedly shown with fMRI in healthy subjects (Rypma and D'Esposito, 1999; Rypma et al., 1999; Wolf and Walter, 2005). With low task demands, brain activation during cognitive processing may be similar in HD patients and controls, or may augment as a consequence of increased cognitive effort to optimize task performance. Given the possible load-dependency of neural dysfunction in clinical HD, parametric activation paradigms may be more suitable for assessment of gradual brain activation and related functional connectivity changes between cortical regions and the striatum (Wolf et al., 2008b).

### Are functional brain activation changes in clinical HD and PSC subjects sensitive and reliable biomarkers of neurodegeneration and disease progression?

The role of fMRI in tracking disease progression needs further evaluation at present. For instance, volumetric data suggest a relative decrease of striatal GM in PSC (Kipps et al., 2005) and cortical volume changes in HD patients over time (Ruocco et al., 2008) however longitudinal fMRI data in both PSC and HD patients are lacking so far. Thus, it is unclear at present if functional or volumetric data are equally sensitive in the detection of early abnormalities in PSC and for tracking disease progression. Moreover, although inferences can be made using cross-sectional years to clinical onset data (Paulsen et al., 2004; Wolf et al., 2007; Zimbelman et al., 2007; Saft et al., 2008), it is still not possible to determine the temporal dynamics of brain activation changes starting from the very early symptomatic period to overtly manifest HD. In designing longitudinal fMRI studies of HD, certain clinical and methodological issues should be taken into account. First, phenotypic variability in HD could represent a potential confound, which may reduce the power of fMRI designs using cognitive paradigms. Moreover, although a number of studies have shown cognitive deficits in PSC subjects (Lawrence et al., 1996; Snowden et al., 2002; Lemiere et al., 2004), the variability in cognitive performance has also been emphasized (Lundervold and Reinvang, 1995). Cognitive impairment does not evolve uniformly in PSC, either over time or with respect to specific cognitive domains (Lemiere et al., 2004), again suggesting a neurobiological and phenotypic variability which has to be taken into account in future longitudinal studies. Studies of multiple cognitive domains (e.g. psychomotor speed, attention, memory and executive function) in populations using short, robust cognitive activation paradigms may offer a unique opportunity to validate a range of stimulation tasks. Those could be used in clinical HD exhibiting manifest cognitive impairment and PSC individuals with subtle cognitive deficits below the clinical threshold and associated neural abnormalities in one or more cognitive domains. Second, practice and learning effects or other strategic factors inherent in given cognitive tasks must be considered as they may be responsible for substantial inter- and intra-individual differences in cognitive capacity (Rypma and D'Esposito, 1999; Rypma et al., 2002). Of note, inferring neural dysfunction in pre-HD so far has been derived from group activation maps, and there is no clear consensus at present how data from complex cognitive activation tasks should be treated at the individual level. Furthermore, methodological heterogeneity in analyzing fMRI data might limit the comparability between studies to a certain extent. While both ROI-driven approaches and whole-brain analyses have been employed, most studies relied on a priori models for the estimation of the hemodynamic response in the context of task-induced stimulation blocks or events. In this context, multivariate statistical analyses might yield more robust results than those obtained by a general linear model approach (Calhoun et al., 2008b). However, these increasingly used analysis and modeling techniques for fMRI data clearly need further evaluation before being implemented in a clinical trial design.

## Diffusion weighted imaging

This method measures the diffusion of water molecules, which is influenced by the fiber architecture of the WM. It allows a voxel-by-voxel comparison of diffusion properties, mostly fractional anisotropy (FA) and mean diffusivity (Beaulieu, 2002). It also permits the tracking of WM fiber connections between different regions of the brain (Mori et al., 1999). A recent review (Bohanna et al., 2008) provides an up-to-date overview of findings in HD. Most consistently reported findings include increases in FA in the putamen and globus pallidum.

It is not straightforward to conclude a specific histological correlate of such FA changes. FA is a measure of the anisotropy of the diffusion and ranges from zero to 1. FA is highest (anisotropic diffusion) in the large fiber tracts such as the cortico-spinal tract but lower where fibers from different directions are crossing or within the GM. It effectively reflects the coherence of the WM. Studies on the histological basis of FA changes usually focused on wallerian degeneration following traumatic brain injury or normal brain development while very little is available on neurodegenerative diseases (see Beaulieu, 2002; Mori and Zhang, 2006 for an overview). It is likely that a degeneration of fibers is associated with a decrease in FA. However, in regions where two fiber tracts are crossing a degeneration of one fiber tract could lead to an increase in FA because the unaffected tract will now dominate and increase WM coherence.

It should also be kept in mind that some of the observed FA changes (Rosas et al., 2006; Klöppel et al., 2008) could be the effect of misregistration of the images in the presence of neurodegeneration. The WM/GM boundary of the putamen could shift inwards. Areas where HD gene carriers already show WM where the FA is relatively high would be compared to overlapping GM in controls. This could lead to the observed FA increases in the putamen. A similar effect could explain reported decreases in FA in the external capsule. An inward shift of the insular cortex could lead to areas where insular cortex in HD gene carries could overlap with WM in controls. Given that the shift of the boundary correlated with disease progression so would FA values and progression. While this mechanism could explain part of the data it certainly does not explain the complete picture. For example, no FA changes have been found in the caudate which should be equally affected by such misregistration. Interestingly, studies looking at mean diffusivity found changes also in the caudate nucleus (Mascalchi et al., 2004; Seppi et al., 2006) which again argues for region specific degenerative changes.

As with fMRI, DWI can potentially be useful when preparing or conducting clinical trials. Part of the substantial clinical variability seen in HD may be a reflection of differential involvement of fiber connections between cortical and subcortical regions. For the case of eye-movements, previous work has already shown a correlation between the individual pattern of fiber connections and the level of impairment in voluntary-guided saccades (Klöppel et al., 2008). New implementations of fiber tracking have been used to study whole circuits with encouraging reproducibility (Draganski et al., 2008).

While few longitudinal data on DWI are available, encouraging results on the exchangeability of DWI data between imaging centers has been presented (Landman et al., 2007) (but see Ozturk et al., 2008).

It is still too early to decide on the usefulness of DWI to either understand the pathophysiology of HD or in the preparation of treatment trials. Currently available data suggest that it can help understanding WM changes already seen in T1-weighted images. When trying to separate PSC from controls using either DWI or T1-weighted imaging, a better separation was achieved using DWI (Klöppel et al., 2008, 2009). A direct comparison is however made difficult as acquisition time was shorter for T1-weighted imaging and because only DWI-weighted data were acquired at a single imaging center. The comparison does however indicate substantial and disease specific changes already in the pre-symptomatic stage of HD to be detectable by DWI.

## Imaging: from mice to man

The identification of the genetic mutation causing HD has led to the development of several animal models of the disease, which have provided fundamental insights into key aspects of disease pathogenesis (Sipione and Cattaneo, 2001; Ramaswamy et al., 2007). Imaging in primates has so far been limited to chemically generated HD models (Roitberg et al., 2002) which are unlikely to represent all aspects of the disease. While a transgenic HD model in primates has been reported very recently (Yang et al., 2008), most imaging research has focused on mouse models (Ramaswamy et al., 2007) and this includes treatment trials (Ferrante et al., 2002). Among other things, these models differ in the number of CAG repeats and whether the genetic mutation replaces a wild-type copy of the huntingtin gene (knock-in mice) or has been inserted into the mouse genome, in addition to two normal copies of the mouse HD gene (transgenic mice) (Ramaswamy et al., 2007). Although the knock-in mouse should represent a better model of HD in terms of genetics, even in the most rapidly progressing knock-in models end-stage disease does not occur until close to the mouse lifespan (∼2 years) which makes studies using these mice challenging (Wheeler et al., 2000; Lin et al., 2001). However, there is a clear effect of repeat length as more CAG repeats cause substantially more degeneration and clinical signs in both transgenic and knock-in mice (Van Raamsdonk et al., 2007).

Imaging studies have focused on the YAC and the R6/2 mouse models of HD (Ramaswamy et al., 2007). Motor symptoms and neuropathological changes in the YAC128 mouse with 128 CAG repeats resemble those of humans. The phenotype of the YAC128 mouse includes a hyperactive phase at the age of 3 months but converts to a hypokinetic phenotype at 6 months compared to wild-type littermates (Slow et al., 2003). Interestingly, testing batteries have found that cognitive decline precedes detectable neuropathology as well as motor signs by the age of 2 months (Van Raamsdonk et al., 2005). So far, the earliest imaging studies in YAC128 mice have been carried out at the later time point of 8 months (Lerch et al., 2008b). Imaging in mice is usually done with a far higher field strength than used in clinical work (7 T and higher in mice vs. 1.5 or 3 T in humans) and scanning times of several hours (compared to around 10 min in humans). At this stage of disease development, comparable to early stage HD in humans, studies report evidence for an enlargement of cortical structures including the right sensorimotor cortex (Lerch et al., 2008b). The finding has been repeated with analysis of cortical thickness (Lerch et al., 2008a). An increase of volume or cortical thickness was also found bilaterally in the cerebellum, while entorhinal cortex and frontal cortex were enlarged on the left. The study also found decreased volumes of the left inferior colliculus and cerebral peduncle as well as right thalamus and striatum, frontal cortex, paraflocculus and anterior commissure (Lerch et al., 2008b).

Comparison of the findings of imaging studies of the YAC128 mouse model with those in humans reveals a number of similarities: most obviously, striatal degeneration is consistently reported in humans (Thieben et al., 2002; Aylward et al., 2004; Kassubek et al., 2004b; Douaud et al., 2006; Paulsen et al., 2006b). More interestingly, mouse models provide new evidence for cortical enlargement which, as reviewed, has also been suggested in humans (Paulsen et al., 2006b). However, while increased cortical volume has been suggested as the result of abnormal development, data from the YAC mouse show a negative correlation between decreasing striatal volume and increasing sensorimotor cortical thickness. Although this could both be a primary effect of the disease, a compensatory mechanism seems more likely.

Rosas and colleagues (2002, 2005, 2008) have conducted a number of studies on cortical thickness in human HD subjects. While increases in cortical thickness and volume were found in the 8-month-old YAC128 mouse (Lerch et al., 2008a), reflecting an early stage of HD, increased thickness in humans was restricted to cingulate areas, with thinning in sensorimotor cortex (Rosas et al., 2008).

Manual outlining and VBM have recently been used to further characterize 18-week-old R6/2 mice with 277 CAG repeats (Sawiak et al., 2009a,b) and produced partially different results. The authors used VBM and located differences in a number of structures including cortex, cerebellum and striatum with gene status without characterizing the directionality. Manual outlining performed by the group found a significantly smaller cortex and striatum in the R6/2 mice while these mice showed a larger globus pallidus. Studies in humans had indicated reduced volume of this structure.

Those discrepancies could well be attributed to differences between mice and men in brain structure as well as to differences in the underlying genetic model. They underline current difficulties transferring results from mouse models to humans. Improved animal models, possibly including primates, are required, particularly as many drugs are tested in the pre-clinical phase in such models before proceeding to human phase 1 and 2 trials.

## The usefulness of imaging as a biomarker

### Biomarkers in HD

A biomarker is “a characteristic that is objectively measured and evaluated as an indicator of normal biological processes, pathogenic processes, or pharmacological responses to a therapeutic intervention” (Biomarkers Definitions Working Group, 2001). It can be used for diagnosis or staging of disease, as an index of disease progression, or to monitor clinical responses to an intervention. In the case of HD, the genetic mutation is known and this therefore serves as a diagnostic marker for the disease. A useful marker for progression in HD needs to be sensitive to the early stages of the disease, be able to distinguish symptomatic benefit from slowing progression, be associated with a pathogenic process and clinical manifestation of the disease, and be measurable objectively and reliably. In other words it must give a clear picture of part of the disease process. It is then assumed that disease-modifying treatments affecting the clinical characteristics and pathogenesis of a disease, leading to clinical benefit, will similarly effect changes in the biomarker thus leading to a reliable marker of treatment efficacy. In addition, to be useful in a typical clinical trial context, a biomarker needs to be tolerated, non-invasive, reliable and reproducible in multi-center settings.

Markers that can predict clinical benefits may potentially also serve as surrogate endpoints. Currently typical clinical endpoints in HD are motor onset and death, but trials in which these outcomes were used to assess drug efficacy would take decades. A surrogate endpoint that predicts motor onset (or death) could be used instead, perhaps allowing therapeutic testing in the asymptomatic years prior to motor onset, and also reducing the length of clinical trials.

However, biomarkers may not always respond to treatment in a predictable manner, as was seen in a recent study for a potential treatment for Alzheimer's disease (AD) in which whole-brain atrophy rates actually increased in patients who responded to an antibody, although at the same time cognitive performance improved slightly (Fox et al., 2005). This finding suggests that therapeutic effects may need to be evaluated using a number of different biomarkers simultaneously, in order to provide a more comprehensive picture of the relationships between changes in different modalities.

### Imaging as a biomarker

MRI measures appear to have many advantages over other measures such as clinical or cognitive scores as a potential biomarker. Brain volume is unaffected by subject mood or tiredness. MRI can be analyzed blind to subject identity and gene status, and if necessary data collected over a number of sites can be analyzed by a single investigator or fully automated and so measures can be objective and highly reproducible (Aylward, 2007; Stonnington et al., 2008). However, brain volume can be affected by medication or co-morbidity, as well as nutritional factors such as hydration (Duning et al., 2005) and so some inter- and intra-individual variability is likely to remain as a result of factors such as these.

Technical issues such as scanner type and consistency can also affect scan measurements. If scans are acquired over a period of time it is important to ensure that changes in scanner calibration have not biased measurements, although some changes (e.g. in voxel size) can be corrected during post-processing (Whitwell et al., 2004). In addition, subjects need to remain still in the scanner for adequate scan quality, which means that in a disease such as HD some subjects will be unsuitable for scanning, and studies will tend to focus on PSC or early affected subjects. Longitudinal studies may risk dropout from the more affected subjects or only be representative of those with less severe motor problems.

### Imaging biomarkers in HD

In HD, caudate volume has been suggested as a potential biomarker, because it can be measured objectively, is associated with the major site of pathology in the disease, and predicts motor onset (Aylward, 2007). Whole-brain atrophy rate has also been suggested, as it is increased in early HD and measured using a robust, semi-automated technique (Henley et al., 2006). Others have pointed out the potential utility of techniques such as VBM (Douaud et al., 2006) and cortical thickness measurements in providing indexes of change over time (Rosas et al., 2008). Only one study has used imaging in a treatment trial (Puri et al., 2002). While the study showed a treatment effect in both imaging and clinical ratings, it should be noted that the number of subjects in each treatment arm was very low (*n*=4 and *n*=3, respectively) and that imaging in late stage HD is very challenging.

Recently, multivariate methods combining information from a number of characteristic degenerative changes have caused increasing interest. While these methods are used primarily to detect subtle degeneration in PSC (Klöppel et al., 2009), they could prove useful to rate individual levels of degenerative change before inclusion in a treatment trial (Vemuri et al., 2008). Their usefulness as a sensitive tool to detect disease progression remains to be shown.

Bohanna et al. (2008) suggested multimodal imaging with the idea that structural MRI would be most sensitive to slow degenerative processes while fMRI could aid in monitoring pharmacodynamics. They also suggested a combination with DWI to test for microstructural changes (Bohanna et al., 2008). While this approach constitutes a methodological challenge, it could facilitate the interpretation of results. As described for the case of AD (Fox et al., 2005), increasing atrophy rates do not necessarily represent increasing neurodegeneration. Similarly, a “normalization” of compensatory hyperactivation in HD found by fMRI could mean a true normalization, but could also mean continuing degeneration.

Another issue, which is relevant to all potential markers of HD, is that motor onset is itself subjective and does not fully reflect the insidious onset of symptoms across multiple domains, which is now known to occur in HD. An ideal surrogate endpoint should fully predict existing clinical outcomes, but this will depend on how well the existing clinical outcome measures reflect the disease in the first place, as well as the variability of the surrogate measure itself.

It is clear that more work is needed to measure change in the natural course of HD, and to determine better indicators of onset and progression of the disease. This is particularly true for PSC and early affected subjects, those who are most likely to enter clinical trials and benefit from disease-modifying treatments at an earlier stage in the disease process. A number of relatively small treatment trials using imaging as an outcome measure are currently being carried out. In addition, large multi-center studies such as TRACK-HD (http://www.track-hd.net) and PREDICT-HD (Paulsen et al., 2008) are ongoing and are likely to provide new insights into both the pathophysiology of HD and its measurement.
